# Association of clinically relevant carpal tunnel syndrome with type of work and level of education: a general-population study

**DOI:** 10.1038/s41598-021-99242-8

**Published:** 2021-10-06

**Authors:** Kamelia Möllestam, Martin Englund, Isam Atroshi

**Affiliations:** 1 Department of Clinical Sciences Lund – Orthopedics, Lund University, Lund, Sweden; 2 Department of Orthopedics, Hässleholm-Kristianstad Hospitals, 28125 Hässleholm, Sweden

**Keywords:** Diseases, Medical research, Risk factors

## Abstract

Carpal tunnel syndrome (CTS) is a common cause of work disability. The association with occupational load and education level has not been established in general-population studies. The purpose of this study was to investigate the association of clinically relevant CTS with work and education. From the Healthcare Register of Skane region (population 1.2 million) in southern Sweden we identified all individuals, aged 17–57 years, with first-time physician-made CTS diagnosis during 2004–2008. For each case we randomly sampled 4 referents, without a CTS diagnosis, from the general population matched by sex, age, and residence. We retrieved data about work and education from the national database. The study comprised 5456 individuals (73% women) with CTS and 21,667 referents. We found a significant association between physician-diagnosed CTS and type of work and level of education in both women and men. Compared with white-collar workers, the odds ratio (OR) for CTS among blue-collar workers was 1.67 (95% CI 1.54–1.81) and compared with light work, OR in light-moderate work was 1.37 (1.26–1.50), moderate work 1.70 (1.51–1.91), and heavy manual labor 1.96 (1.75–2.20). Compared with low-level education, OR for CTS in intermediate level was 0.82 (0.76–0.89) and high-level 0.48 (0.44–0.53). In women and men there is significant association with a dose–response pattern between clinically relevant CTS and increasing manual work load and lower education level. These findings could be important in design and implementation of preventive measures.

## Introduction

Carpal tunnel syndrome (CTS) is a frequent cause of hand symptoms and activity limitations, most commonly affecting people of working age^1, 2^. The potential association between the occurrence of CTS and work-related factors has been extensively debated. Most previous studies that investigated the etiology of CTS have concluded that CTS is mainly associated with individual risk factors, such as higher age, female sex, obesity, rheumatoid arthritis, diabetes mellitus, hypothyroidism, acromegaly, pregnancy and trauma^3, 4^. However, several studies have suggested an important relationship between work and CTS^5^. Occupational activities, such as forceful manual work, highly repetitive flexion and extension of the wrist, extreme wrist postures, and use of vibratory tools, may lead to an increased risk of CTS^3–12^. High prevalence has been shown in certain occupational groups, for example those working with assembly, food processing, packaging, particularly in industries and cold conditions^3, 13, 14^. Previous research has mostly involved specific occupational cohorts and used various case definitions for CTS, not always based on diagnosis made by physicians. Other studies have included only surgical cohorts and thus the findings regarding associations would only apply to surgically treated CTS. To our knowledge, no previous studies assessing the association between CTS and work have involved all individuals in a general population who have sought health care for hand problems that were diagnosed by a physician as CTS^15, 16^. As opposed to the large interest in the relationship with work, little is known about the possible association between CTS and level of education. Although one previous population-based study found a possible association between level of education and having undergone surgery for CTS, no studies have addressed the association with all clinically relevant CTS^17^.

## Methods

The aim of this case–control study based on a general population was to investigate the association between clinically relevant CTS and type of work and level of education.

### Data collection

Using the Skane Healthcare Register (SHR), we identified new cases with physician-diagnosed CTS in the population of Skane region in southern Sweden (1.2 million inhabitants, one-eighth of the population of Sweden). All inpatient and outpatient healthcare provided in the region, including primary care, is registered in the SHR. Diagnoses are chosen and registered by the doctors according to the International Classification of Diseases and Related Health Problems 10 (ICD-10) system. From the SHR, we retrieved data on all subjects who were given a physician-made primary diagnosis of CTS during a 5-year period (from January 1, 2004 through December 31, 2008). The inclusion criteria were; (1) age at diagnosis within range of 17 years to 57 years, (2) primary diagnosis of CTS made by a medical doctor, and (3) resident in the region during 3 calendar years prior to the date of diagnosis. We excluded subjects who had received a diagnosis of CTS during 3 years before the date of first CTS diagnosis registered in the study period.

For each CTS case we randomly sampled 4 matched referent individuals from the general population. The matching variables were sex, year of birth, and district of residence. The referent individuals had to be residents in the region and had no CTS diagnosis. No other exclusion criteria were used. Therefore, all individuals (the cases and the randomly selected referents) irrespective of their work status were included.

We retrieved data regarding type of work and level of education from a national database, the Longitudinal integrated database for health insurance and labor market studies (LISA). The LISA database is part of Statistics Sweden, and integrates existing data from the labor market, educational sector and social sector, which is updated with annual registers^18^.

### Definitions

We first classified type of work as blue-collar or white-collar based on the Swedish Standard Classification of Occupations (SSYK 96) (Appendix)^19, 20^. We also used a second classification of occupations, based on a previous study by Wolf et al., in which occupations were categorized as light, light-moderate, moderate or heavy manual work^21^. We classified the level of education according to the Swedish Education Classification (SUN): levels 1 and 2 (primary and lower secondary education up to 9 years) are classified as “low”, levels 3 and 4 (upper secondary education 2 or 3 years) as “intermediate”, and levels 5 to 7 (post-secondary education and postgraduate education) as “high”^22^. All definitions were made before data analysis.

### Statistical analysis

We used the Chi-square test to compare the CTS cohort with the referent cohort regarding type of work and level of education, stratified according to sex. We estimated the odds ratios (OR) with 95% confidence intervals (CI) of CTS using three conditional logistic regression models. The first model was to estimate the total effect of education, and thus education but not occupation was included (because occupation is an intermediate in the association between education and CTS). Age and sex did not vary within matched sets and were thus adjusted for by design and use of conditional regression. In two additional models, we estimated the total effect of occupation adjusted for education (because education is a potential confounder of the association between occupation and CTS). A separate model for each of the classifications of occupations was used, one for type of work “blue/white collar” and one for occupational groups “light/light-moderate/moderate/heavy”. These two models included only one occupational variable (ie, the model analyzing “blue/white collar” did not include “occupational group”, and vice versa). A p-value below 0.05 was used for statistical significance. The analyses were performed with STATA v 16.0 (Stata Corporation, College Station, TX).

### Ethical approval

The study was conducted according to the Declaration of Helsinki and was approved by the Ethical Review Board of Lund University (Dnr 2011/432). Need for informed consent was waived by the Ethical Review Board of Lund University. The population in the Skane region was informed of the study via an advertisement in newspapers and were offered the option to “opt-out”, which is a standard process for population-based studies using register data as recommended by the Ethical Review Board.

## Results

### Study cohorts

The study population has been described previously^23^. During the 5 calendar years (2004–2008), a primary physician-made diagnosis of CTS was received by 7108 subjects, aged 17 to 57 years, who were residents of Skane region 3 years prior to the diagnosis. We excluded 1641 individuals because they had received a CTS diagnosis also during the 3 years preceding the date of first diagnosis in the study period and another 11 because no eligible matched referents could be found. The final cohorts comprised 5456 individuals with CTS and 21,667 matched reference individuals from the general population without CTS diagnosis. The mean age for the CTS and general population cohorts was 43 years and 73% were women (Table 1). The diagnosis of CTS was most common in the age group 45–57 years (47% in women and 50% of men).

**Table 1:** Characteristics in the carpal tunnel syndrome (CTS) cohort and the matched reference cohort without CTS.

	Women		Men	
	CTS	Referents	CTS	Referents
	n = 3966	n = 15,756	n = 1490	n = 5911
	n (%)	n (%)	n (%)	n (%)
**Age group**				
17–34	857 (21.6)	3381 (21.5)	308 (20,7)	1215 (20.6)
35–44	1250 (31.5)	4978 (31.6)	436 (29.2)	1728 (29.2)
45–57	1859 (46.9)	7397 (46.9)	746 (50.1)	2968 (50.2)
**Type of work**				
Blue-collar	2045 (51.6)	6351 (40.3)	968 (65.0)	2729 (46.2)
White-collar	1336 (33.7)	7006 (44.5)	317 (21.3)	2192 (37.1)
Unknown^a^	585 (14.8)	2399 (15.2)	205 (13.8)	990 (16.7)
**Occupational group**				
Light manual work	1399 (35.3)	7024 (44.6)	297 (19.9)	2005 (33.9)
Light-moderate	1277 (32.2)	4378 (27.8)	115 (7.7)	581 (9.8)
Moderate	482 (12.2)	1290 (8.2)	163 (10.9)	514 (8.7)
Heavy	223 (5.6)	665 (4.2)	710 (47.7)	1821 (30.8)
Unknown^a^	585 (14.8)	2399 (15.2)	205 (13.8)	990 (16.7)
**Level of education**				
Low	754 (19.0)	2329 (14.8)	406 (27.2)	1169 (19.8)
Intermediate	2193 (55.3)	7767 (49.3)	856 (57.4)	3092 (52.3)
High	988 (24.9)	5542 (34.5)	216 (14.5)	1568 (26.5)
Unknown	31 (0.8)	218 (1.4)	12 (0.8)	82 (1.4)

^a^Includes true missing as well as unemployed, students, disability pension, early retirements, entrepreneurs and project employments.

### Work

Of the women with CTS 52% were blue-collar workers and 34% white-collar workers compared to 40% and 44%, respectively, of the general population referents (p < 0.001) (Table 1). Among the men with CTS 65% were blue-collar workers and 21% white-collar workers compared to 46% and 37%, respectively, of the general population referents (p < 0.001).

Among the women with CTS 35% had light manual work, 32% had light-moderate manual work, 12% had moderate manual work and 6% had heavy manual labor, compared to 45%, 28%, 8% and 4% respectively, of the general population referents (p < 0.001). Among the men with CTS 20% hade light work, 8% had light-moderate work, 11% had moderate work and 48% had heavy manual labor, compared to 34%, 10%, 9% and 31% respectively, of the general population referents (p < 0.001).

### Education

Among women with CTS level of education was classified as low in 19%, intermediate in 55%, and high in 25%, compared to 15%, 49% and 35%, respectively, among the population referents (p < 0.001) (Table 1). Among men level of education was classified as low in 27%, intermediate in 57%, and high in 15%, compared to 20%, 52% and 27% respectively, among the population referents (p < 0.001).

### Association with CTS

Compared with white-collar workers the OR for CTS among blue-collar workers was 1.67 (95% CI 1.54–1.81) (Table 2). Compared with light work, the OR for CTS was 1.37 (95% CI 1.26–1.50) for light-moderate work, 1.70 (95% CI 1.51–1.91) for moderate work, and 1.96 (95% CI 1.75–2.20) for heavy manual labor. Compared with persons with low-level education, the OR for CTS among those with intermediate-level education was 0.82 (95% CI 0.76–0.89) and among those with high-level education was 0.48 (95% CI 0.44–0.53).

**Table 2:** Association between level of education and type of work and carpal tunnel syndrome.

Model	Odds ratio (95% CI)^a^
**Level of education**^**b**^	
Low	Referent
Intermediate	0.82 (0.76–0.89)
High	0.48 (0.44–0.53)
**Type of work**^**c**^	
White-collar	Referent
Blue-collar	1.67 (1.54–1.81)
**Occupational group**^**d**^	
Light manual work	Referent
Light-moderate	1.37 (1.26–1.50)
Moderate	1.70 (1.51–1.91)
Heavy	1.96 (1.75–2.20)

^a^Odds ratios with 95% confidence intervals from conditional logistic regression models; age and sex did not vary within matched sets and thus adjusted for by design and use of conditional regression.

^b^Estimating the effect of education (model does not include type of work or occupational group).

^c^Estimating the effect of type of work (white-collar/blue-collar) adjusted for education (model does not include occupational group variable).

^d^Estimating the effect of occupational group (light/light-moderate/moderate/heavy) adjusted for education (model does not include type of work variable).

## Discussion

Our results show significant relationship between physician-diagnosed CTS and type of work (higher risk in blue-collar vs white-collar) in both women and men. These results are in line with previous studies. In a study from a region in Italy, both female and male blue-collar workers had higher age-specific rates of surgically-treated CTS than white-collar workers at all ages^8^. Another study from Italy showed that blue-collar work is a risk factor for surgically-treated CTS in both women and men^16^. However, the study, besides involving only patients with surgically-treated CTS, was not truly population-based but rather based on random sampling of 20 cases and 40 controls from discharge records of local hospitals.

In a large population-based study in Southern Sweden, the prevalence of CTS among active blue-collar workers was significantly higher than that among white-collar employees even after adjusting for sex, age, and body mass index^1^. The reason for the higher prevalence of CTS among blue-collar workers is believed to be that certain occupational risk factors are more common among blue-collar workers, such as forceful manual work, extreme wrist postures and use of vibratory tools^8^. These theories are in agreement with our results where there is a significant association between heavy manual work and CTS in both women and men. Our findings suggest a dose–response relationship between increasing manual load and CTS, with the heavy occupation group almost at twice the odds of having CTS compared with the referent group.

Our results support those of previous studies regarding the association between occupational load and CTS. A study from Finland investigated the relationship between exposures to different physical load factors and CTS in 6254 individuals aged 30 years or older, and found that hand grip with high forces was related to an increased prevalence of CTS (OR 1.7, 95% CI 1.2–2.5)^15^. Although this study was population-based, subjects were recruited through interviews (performed by nurses) and clinical health examinations, whereas our study population consists of individuals who had sought healthcare for hand problems and were diagnosed with CTS by the treating physician. Two population-based studies from Denmark investigated the association between occupational load and CTS using direct exposure measures and found that rapid wrist movements, measured as wrist angular velocity, and repetitive movements, measured as mean power frequency, were associated with increased risk of CTS (incidence rate ratio 2.31 [95% CI 2.09–2.56] and 1.83 [1.68–1.98], respectively), and that there was a positive association between hand load and incidence rates of CTS^11, 24^. These studies included only hospital-diagnosed CTS and thus all cases treated exclusively in primary care (and possibly in private practice by specialists) were not included. Our study has shown similar findings to these population-based studies that used more precise exposure measures, but with the additional advantage that it included all cases of clinically relevant CTS.

Several previous studies concerning the relationship between work and CTS have been conducted on various industrial cohorts. A 3-year follow-up study of 1107 newly-hired workers in the United States found that forceful gripping (OR 2.59, 95% CI 1.12–5.99) and lifting > 1 kg (OR 3.27, 95% CI 1.27–8.44) were significantly associated with incident CTS^2^. In Italy, a 10-year longitudinal study on a group of industrial and service workers, that classified exposure with respect to action limit and threshold limit values (as proposed by the American Conference of Governmental Industrial Hygienists) found increased incidence of CTS among workers exposed between the action limit and threshold limit values (hazard ratio 2.18, 95% CI 1.86–2.56) and for workers exposed above the threshold limit value (hazard ratio 2.07, 95% CI 1.52–2.81)^12^.

Our results also show significant association between CTS and level of education, with higher risk of physician-diagnosed CTS among individuals with low level of education. Our logistic regression model indicates that the risk of CTS decreases with increasing education level, with the highest education level at less than half the odds of having CTS compared with the referent group. The association between CTS and level of education has been investigated in very few previous studies. A study from Finland, analyzed data from 6256 individuals aged ≥ 30 years who had undergone surgery for CTS, with regard to level of education, BMI, smoking, leisure time physical activity, workplace physical exposures, and medical conditions (diabetes, RA, hypothyroidism and osteoarthritis) and found that individuals with low education were more likely than those with high education to undergo surgery for CTS^17^. A Brazilian study about the characteristics of a retrospective hospital cohort of 150 patients diagnosed with CTS found that more than half had a lower education level than high school^25^.

Individuals with the highest levels of education more often have occupations that less likely involve highly demanding manual work tasks, whereas individuals with lower education are probably more likely to have heavy manual work^26^. Moreover, level of education (and income) usually defines socioeconomic status. The association between low socioeconomic status and inferior health is well established^27, 28^. In addition to the role of education in determining job opportunities (lower education more likely in higher physical exposure jobs), its association with CTS may also be through other mechanisms such as increasing BMI, which is an important risk factor in CTS.

Our study has limitations. First, we identified the individuals with CTS through their health care attendance and it is possible that blue-collar workers with manually high demanding jobs are more likely to seek health care for hand symptoms that cause work limitations. Second, duration of employment was not specified, so the occupational exposure time is unknown. However, previous studies have indicated that even short periods of exposure can be sufficient for developing occupational CTS^5^. We did not use direct measures to determine the level of exposure for each type of work, as it would be difficult to accomplish in a general population study. Our findings however support both those of previous studies performed on workers in specific industries as well as those of previous population-based studies that used direct exposure measures. Furthermore, the data did not permit adjustment for possible confounding factors. For example, obesity is considered an important confounder in some studies^5^. The strengths of our study include the truly population-based design (all incident cases and randomly chosen matched referents from a whole a general population) and CTS of clinically important severity (individuals sought healthcare) but not restricted to surgically treated. Further, the cases were diagnosed with CTS by a medical doctor within usual healthcare at all levels of healthcare including primary care where a substantial proportion of individuals seeking healthcare for CTS are managed.

## Conclusion

Using a large population-based data set we found evidence of a dose–response relationship between both level of education and type of work with the risk of clinically relevant CTS, with increased risk the higher the occupational load and the lower the level of education. These findings could be important in design and implementation of preventive measures.

## Supplementary Information

Supplementary Information.

## Data Availability

The datasets generated and/or analyzed during the current study are not publicly available due to that individual patient privacy could be compromised, but are available from the corresponding author on reasonable request.
