# Supplementary material for: Association of clinically relevant carpal tunnel syndrome with type of work and level of education: a general-population study
Source: Sci Rep. 2021 Oct 6;11:19850. doi: 10.1038/s41598-021-99242-8 (PMC8494938; doi:10.1038/s41598-021-99242-8)
Supplement: Supplementary file 1 — Supplementary Information. [file 41598_2021_99242_MOESM1_ESM.docx]

**Association of Clinically Relevant Carpal Tunnel Syndrome with Type of Work and Level of Education: A General-Population Study**

Authors:

Kamelia Möllestam*, MD^1,2^ (kamelia.mollestam@skane.se)

Martin Englund, PhD^1^ (martin.englund@med.lu.se)

Isam Atroshi, PhD^1,2^ (isam.atroshi@med.lu.se)

^1^ Clinical Epidemiology Unit, Department of Clinical Sciences Lund – Orthopedics, Lund University, Lund, Sweden.

^2^ Department of Orthopedics, Hässleholm-Kristianstad Hospitals, Hässleholm, Sweden.

Corresponding author

Kamelia Möllestam, Department of Orthopedics, Hässleholm-Kristianstad Hospitals

SE-28125 Hässleholm, Sweden

kamelia.mollestam@skane.se, Tel: +46 44 3091273; Fax: +46 44 3091264

**Appendix.** Occupational groups according to the Swedish Standard Classification of Occupations (SSYK 96)

1 Managers

2 Occupations requiring advanced level of higher education

3 Occupations requiring higher education qualifications or equivalent

4 Administration and customer service clerks

5 Service, care and shop sales workers

6 Agricultural, horticultural, forestry and fishery workers

7 Building and manufacturing workers

8 Mechanical manufacturing and transport workers, etc.

9 Elementary occupations

0 Armed forces occupations

Groups 0-4 are classified as white-collar workers, except occupations 4131 (warehouse manager), 4132 (vehicle manager, traffic manager, transport manager etc), 4150 (postman, postal manager etc) and 4213 (casino staff, croupier, dealer), classified as blue-collar.

Groups 5-9 are classified as blue-collar workers, except occupations 5111 (flight attendants), 5113 (guides and tour guides) and 5227 (telemarketer), classified as white-collar.
